# COVID-19 pneumonia: do not leave the corticosteroids behind!

**DOI:** 10.2217/fmb-2020-0199

**Published:** 2021-03-12

**Authors:** Simone Mornese Pinna, Silvia Scabini, Silvia Corcione, Tommaso Lupia, Francesco G De Rosa

**Affiliations:** ^1^Department of Medical Sciences, Infectious Diseases, University of Turin, Turin, Italy

**Keywords:** ARDS, coronavirus, corticosteroids, COVID-19, inhaled corticosteroids, pneumonia, SARS-CoV-2

## Abstract

The host inflammatory response is critical in the progression of lung injuries in patients with SARS-CoV-2. Corticosteroids (CS) have been widely used as immunomodulating agents, but the right timing, dosage and type of molecule are unknown. In fact, the early use of CS could facilitate the viral replication but late administration may not prevent the alveolar damage. Nevertheless, a short administration of high doses of CS in the early stage of the inflammatory phase resulted in favorable outcomes. Noteworthy, some inhaled CS inhibited *in vitro* the viral replication of SARS-CoV-2. We aimed to define the place in therapy for CS in COVID-19 infection describing the features of patients who may benefit from their administration.

In January 2020, SARS-CoV-2 was isolated in Wuhan, China, and spread all over the country and subsequently worldwide, infecting over 16,000.000 people, including >600,000 deaths worldwide (ECDC, 29 July 2020). The clinical manifestations of COVID-19 range from mild symptoms to severe illness with bilateral pneumonia leading to acute respiratory distress syndrome (ARDS) and multiple organ failure [1]. So far, there is a lack of data about efficient treatment strategies for SARS-CoV-2 infection, and management is largely supportive.

Patients presenting with severe manifestations related to COVID-19 were reported to have increased plasma concentrations of chemoattractant molecules and pro-inflammatory cytokines, including IL-10, IL-6, IL-1 and TNF-α [2]. The innate and adaptive immune response largely contributes to the development of a ‘cytokine storm’, playing a pivotal role throughout the development and progression of ARDS. In agreement with this, patients with severe COVID-19 presented high levels of d-dimer, serum ferritin, IL-6 and C-reactive protein [2]. Treatment strategies are focused on antivirals and immunomodulating agents differently combined.

Corticosteroids (CS), because of their anti-inflammatory properties, have been widely used among patients with SARS-CoV and MERS-CoV with contrasting results [3–8]. However, most of the data came from observational studies where CS were used in different doses. Furthermore, also the timing of CS administration may be crucial for the efficacy of CS treatment. In fact, some patients received CS very early after diagnosis, others received CS as a ‘rescue-therapy’ when other interventions failed [4,5,9,10].

Several studies reporting increased mortality following CS administration were carried out in intensive care unit setting, usually in patients severely compromised or with pre-existing underlying comorbidities [4,5,9]. Based on available data on steroids for the treatment of SARS and MERS-infected patients, early evidence was against the use of CS and only weak recommendation based on low-quality evidence supported the use of CS for patients with COVID-19 [11–13].

## Systemic CS in COVID-19

The pathogenesis of SARS-CoV-2 infection has been proposed to occur during three phases. Following inoculation, SARS-CoV-2 can rapidly spread and replicate within the epithelial cells of the respiratory tract or small intestine causing mild and nonspecific symptoms (first phase). In the second phase, SARS-CoV-2 infection spreads to the lungs resulting in pneumonia characterized by bilateral pulmonary infiltrates on chest imaging and worsening of clinical symptoms with fever, cough, and in some cases, hypoxia. A little subgroup of patients with COVID-19 related pneumonia develops massive systemic inflammation, due to the upregulation of several pro-inflammatory and procoagulant cytokines leading to increased pulmonary vascular permeability, thrombotic disorders, severe respiratory failure and higher mortality rates (Phase III) [2,14]. Inflammatory disorder and cytokine expression in severe COVID-19 manifestation are comparable to that observed in ARDS [15]. CS influence different inflammatory pathways, demonstrating to reduce mortality in patients with ARDS [16]. However, in order to be effective, CS should be started early following the ARDS development [17].

- The RECOVERY trial has demonstrated that once-daily administration of 6 mg of dexamethasone was associated with reduced mortality compared with patients treated with standard of care (29.3 vs 41.4% respectively) [18];
- In a retrospective analysis of 201 individuals with COVID-19 pneumonia, patients who developed. ARDS, had lower mortality if methylprednisolone was added to the standard of care. Methylprednisolone at 1–2 mg/kg/d was also associated with faster recover of Sp02 in 46 patients with mild COVID-19 manifestations [19];
- Early use of CS displayed reduced exposure to invasive mechanical ventilation, intensive care unit admission and overall mortality in a multi-center study by Fadel *et al.* [20];
- Recently, a recent meta-analysis of seven randomized clinical trials evaluating the efficacy of CS in critically ill patients with COVID-19, found reduced mortality among 678 individuals who received CS than in 1025 patients who were not (p < 0.01) [21]. The pooled analysis from this study has offered evidence that survival rates have improved in critically ill patients with COVID-19 treated with CS [21]. Similar results were observed even in our series of COVID-19 patients (data not yet published);
- Following the results from the RECOVERY trial and other evidence about the efficacy of CS in severe COVID-19, including patients with ARDS, now recommendations from the main guidelines have been revised and now CS are recommended for the treatment of patients with COVID-19 and severe or moderate manifestation by requiring supplemental oxygen [18,22,23].

We believe that patient selection plays a pivotal role in the success of systemic CS administration. In fact, the very early administration of systemic CS in patients with a stable mild disease, for example, without signs of respiratory worsening or need for high-flow oxygen supply and constantly low-grade inflammation established by markers such as C-reactive protein, D-dimer, TNF and, fibrinogen might favor the first viral phase, resulting in high viral replication and eventually precipitating the subsequent phases. On the other hand, CS administration late in the course of the inflammatory stage, once the immune system activation has been established, could be unhelpful to prevent lung injuries. Thus, the effort should be directed to pinpoint the threshold at which a patient could benefit from CS administration. Progressive respiratory illness in COVID-19 is associated with marked elevation of inflammatory markers such as CRP, PCT, ferritin and D-dimer [18,24]. Persistent fever is a common clinical sign of systemic inflammation and has been associated to the development of respiratory failure and ARDS in COVID-19 [25]. Thus, based on these findings, we tried to identify simple and reliable markers helping to select early in the course of COVID-19 those patients that could benefit from systemic CS administration (Table 1). The goal of early CS treatment is to prevent lung damage induced by the cytokine storm associated with the development of ARDS. The evidence of CS on ARDS, regardless of the underlying cause has been estimated by a recent meta-analysis of Ye *et al.* [13].

**Table 1.** Proposed criteria to introduce systemic corticosteroids in the treatment of SARS-CoV-2 infection.

O_2_ supply requirement and ≥1 of the following criteria:
• Patient has dyspnea at rest or during minimal activity (sitting up in bed, standing, talking)
• Breathing rate >30 bpm
• PaO_2_ <60 mmHg in ambient air at admission or worsening hypoxemia during hospitalization
• Persistent fever (body temperature >38°C)
• Radiographic progression of lung opacities
• CRP mg/l >100
• Ferritin μg/l >1000

BPM: Beats per minute; CRP: C-reactive protein; O_2_: Oxygen; PaO_2_: Partial pressure of oxygen.

## Inhaled CS in COVID-19

Early in the pandemic, physicians were expected that people affected by asthma or structural lung diseases, such as chronic obstructive pulmonary disease (COPD), would have been more susceptible than others to severe manifestations. However, the prevalence of COPD in patients with COVID-19 is below 3% [2,26].

In patients with COPD innate and adaptive immune response to respiratory infections is impaired, and *in vitro* data suggest that the production of inflammatory cytokines in response to infective agents could be reduced. Another possibility is that some medications taken by patients with COPD might reduce the risk of infection. Inhaled CS (ICS) with or without β2-agonist are the mainstay of treatment in asthma and COPD [27]. Ciclesonide (CIC) and mometasone, two ICS used in asthma and allergic rhinitis have been found to significantly inhibit replication of SARS-COV-2 and other coronaviruses *in vitro*. The association of immunomodulation and antiviral effect, combined with the low rate of serious adverse events, could be useful for patients with COVID-19, presenting with mild symptoms, to reduce the risk of progression to severe manifestations. Yamaya *et al.* found that glycopyrronium, formoterol, and budesonide reduced the viral replication of HCoV-229E on airway epithelial cells [28].

Clinical data about the effectiveness of ICS in patients with COVID-19 is limited and anecdotical [29]. The efficacy and safety of CIC and budesonide/formoterol in patients with mild COVID-19 are currently under investigation in two RCT (ClinicalTrials.gov identifier: NCT04330586, NCT04377711, NCT04416399, NCT04355637, NCT04331470 NCT04331054) and will contribute to elucidate the answer.

**Figure 1.**
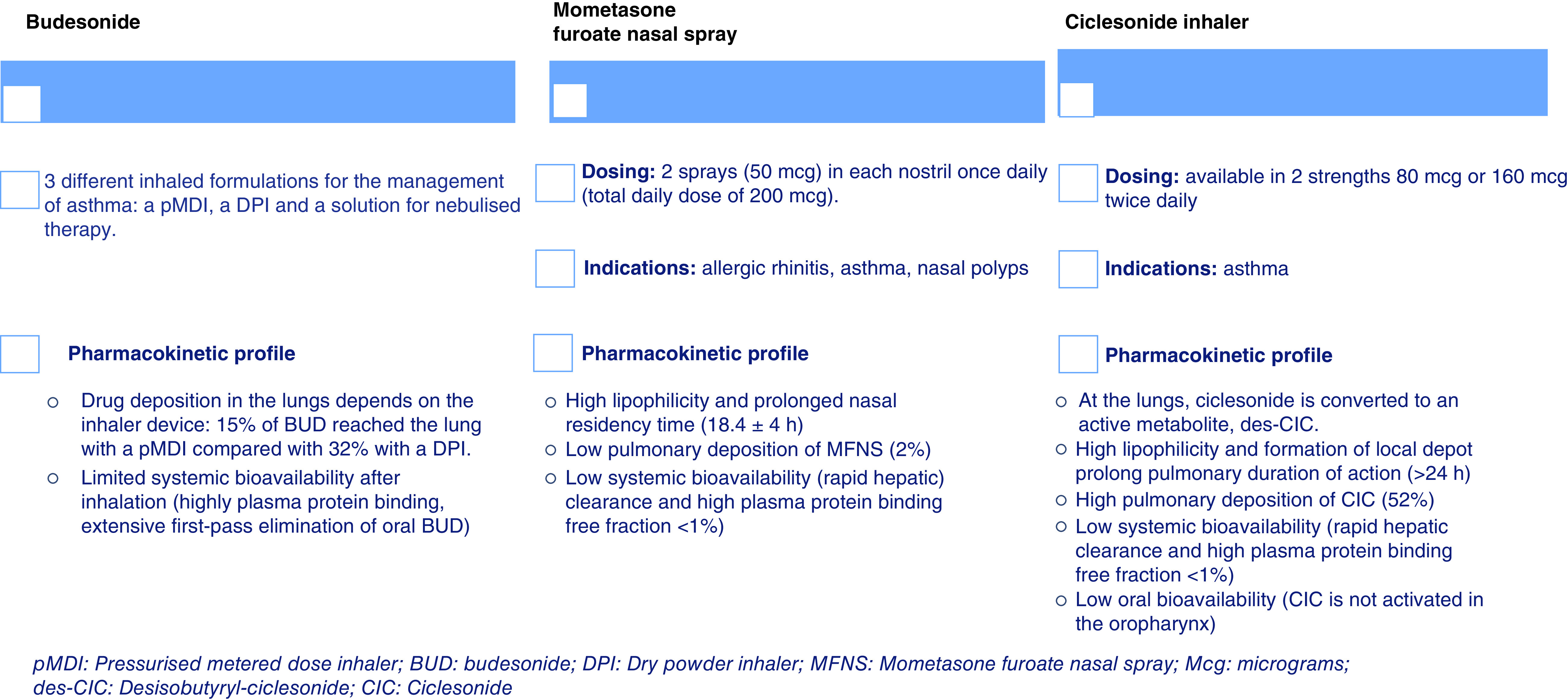
Main characteristics of inhaled corticosteroids with effect against SARS-COV-2. BUD: Budesonide; CIC: Ciclesonide; des-CIC: Desisobutyryl-ciclesonide; DPi: Dry powder inhaler; Mcg: Micrograms; MFNS: Mometasone furoate nasal spray; pMDI: Pressurized metered dose inhaler.

We report the features of the main inhaled CS with effect against SARS-CoV-2 (Figure 1). The role of ICS against COVID-19 is still under investigation, but we should consider their potential because of their antiviral effect *in vitro*, particularly for molecules with the highest potential such as CIC and mometasone. Concerning to the characteristics of ICS described above, the ideal timing of administration would be in an earlier stage compared with systemic CS, in other words when SARS-CoV-2 is in the first replicative phase. Here we tried to elucidate the characteristics of patients in the early replicative phase of COVID-19, that could benefit from ICS administration (Table 2).

**Table 2.** New parameters proposed to identify patients that may benefit from inhaled steroids in COVID-19.

Inclusion criteria	Exclusion criteria
Hospitalized with ≥1:	Patients taking chronic medications known to prolong QT interval
- No evidence of diffuse bilateral infiltrates on chest x-ray or CT scan	O_2_ demand >5 l/min
- Cough or shortness of breath	Contraindications/hypersensitivity to treatment with one or more agents.
- Sp02 <94% in room air	ICU admission
- Requiring O_2_ supply with nasal cannulas or facial mask (not exceeding 5 l/min)	

ICU: Intensive care unit; O_2_: Oxygen; SpO_2_: Oxygen saturation.

## Future perspective

The emergence of SARS-CoV-2 infection requires urgently new therapeutic options. Currently, evidence on the effectiveness of CS came from observational, retrospective series and a recent randomized controlled trial. Systemic CS are undoubtedly molecules suffering some complications and their use should be evaluated carefully. CS could help to reduce lung injuries in patients rapidly progressing to ARDS with signs of systemic inflammatory response and requiring increasing demand for oxygen. Conversely, in the viral and early phase of infection, characterized by mild respiratory symptoms, the use of systemic CS could precipitate hypoxemia and respiratory failure.

On the other hand, inhaled CS could find their rationale in the treatment of COVID-19 by the evidence that SARS-CoV-2 replicates early in the upper respiratory tract including nasal epithelium, trachea, bronchi and only subsequently in lungs [30]. Probably, CIC is a good candidate for its antiviral properties and for its restricted anti-inflammatory activity to the desired site of action, since it is converted to its pharmacologically active metabolite at the lungs. Considering also its low oral bioavailability, high hepatic clearance and plasma protein binding, systemic exposure is very low, resulting in fewer side effects, such as adrenal suppression. The early administration of inhaled CS with direct antiviral activity in addition to immunomodulatory effect might be considered in patients with SARS-CoV-2 infection even without asthma or COPD presenting with COVID-19 pneumonia with mild oxygen demand.

There is now strong evidence that CS are associated with improved survival among critically ill patients with COVID-19 in both mechanically ventilated or receiving other types of oxygen supply. For the first time, based on the current evidence available, we tried to elucidate simple criteria helping to identify the exact threshold at which inhaled and systemic CS could be started in patients with COVID-19. Further studies and clinical trials exploring the role of inhaled and systemic CS will definitively provide an answer to elucidate the timing of systemic CS administration and the role of inhaled steroids in the treatment of COVID-19.

**Executive summary**

**Background**

- Glucocorticoids are hormones that regulate a wide range of physiologic activities and are essential for life. They are widely used in medicine due to their profound immune-modulatory actions by decreasing the inflammatory response.

**Systemic corticosteroids**

- Systemic corticosteroids (CS): efficacy and safety of CS in COVID-19 is based on previous experience with other coronaviruses.
- CS have reduced the mortality of patients with COVID-19 and sever respiratory failure but the timing of administration, is unclear.
- CS should be administered early at the onset of respiratory failure when inflammatory markers rapidly deteriorate.

**Inhaled CS**

- Inhaled CS are the mainstay of the treatment of asthma while systemic CS are used for a variety of inflammatory conditions.
- *In vitro* studies with budesonide, in combination with glycopyrronium and formoterol, demonstrated to inhibit replication and cytokine production against some strains of coronavirus.
- Ciclesonide *in vitro* blocks SARS-CoV-2 ribonucleic acid replication and inhibits SARS-CoV-2 cytopathic activity.
- Patients with non-severe manifestation of COVID-19 might benefit from inhaled CS by directly reducing the viral load and modulating the inflammation in the upper airways.

**Future perspective**

- CS have been shown to reduce mortality in SARS-CoV-2 infection and to reduce viral load in the first airways, *in vitro*, however it will be essential to identify valid criteria to pinpoint the correct timing of administration and therefore achieve the maximum benefit from the administration of these molecules.

## References

[1] Wu C, Chen X, Cai Y Risk factors associated with acute respiratory distress syndrome and death in patients with coronavirus disease 2019 pneumonia in Wuhan, China. JAMA Intern. Med. 180(7), 934–943 (2020). 32167524 10.1001/jamainternmed.2020.0994 PMC7070509

[2] Zhou F, Yu T, Du R Clinical course and risk factors for mortality of adult inpatients with COVID-19 in Wuhan, China: a retrospective cohort study. Lancet 395(10229), 1054–1062 (2020). 32171076 10.1016/S0140-6736(20)30566-3 PMC7270627

[3] Lee N, Allen Chan KC, Hui DS Effects of early corticosteroid treatment on plasma SARS-associated Coronavirus RNA concentrations in adult patients. J. Clin. Virol. 31(4), 304–309 (2004). 15494274 10.1016/j.jcv.2004.07.006 PMC7108318

[4] Arabi YM, Mandourah Y, Al-Hameed F Corticosteroid therapy for critically ill patients with middle east respiratory syndrome. Am. J. Respir. Crit. Care Med. 197(6), 757–767 (2018). 29161116 10.1164/rccm.201706-1172OC

[5] Khalid I, Alraddadi BM, Dairi Y Acute management and long-term survival among subjects with severe middle east respiratory syndrome coronavirus pneumonia and ARDS. Respir. Care 61(3), 340–348 (2016). 26701365 10.4187/respcare.04325

[6] Zhao Z, Zhang F, Xu M Description and clinical treatment of an early outbreak of severe acute respiratory syndrome (SARS) in Guangzhou, PR China. J. Med. Microbiol. 52(8), 715–720 (2003). 12867568 10.1099/jmm.0.05320-0

[7] Lee N, Allen Chan KC, Hui DS Effects of early corticosteroid treatment on plasma SARS-associated Coronavirus RNA concentrations in adult patients. J. Clin. Virol. 31(4), 304–309 (2004). 15494274 10.1016/j.jcv.2004.07.006 PMC7108318

[8] Alfaraj SH, Al-Tawfiq JA, Assiri AY, Alzahrani NA, Alanazi AA, Memish ZA. Clinical predictors of mortality of Middle East Respiratory Syndrome Coronavirus (MERS-CoV) infection: a cohort study. Travel Med. Infect. Dis. 29, 48–50 (2019). 30872071 10.1016/j.tmaid.2019.03.004 PMC7110962

[9] Arabi YM, Arifi AA, Balkhy HH Clinical course and outcomes of critically ill patients with middle east respiratory syndrome coronavirus infection. Ann. Intern. Med. 160(6), 389–397 (2014). 24474051 10.7326/M13-2486

[10] Hsu L, Lee C, Green JA Clinical features of index patient and initial contacts. Emerg. Infect. Dis. 9(6), 713–717 (2003). 12781012 10.3201/eid0906.030264 PMC3000162

[11] WHO. Clinical management of severe acute respiratory infection when novel coronavirus (nCoV) infection is suspected. (2020). https://www.who.int/publications-detail/clinical-management-of-severe-acute-respiratory-infection-when-novel-coronavirus-(ncov)-infection-is-suspected

[12] Russell CD, Millar JE, Baillie JK. Clinical evidence does not support corticosteroid treatment for 2019-nCoV lung injury. Lancet 395(10223), 473–475 (2020). 32043983 10.1016/S0140-6736(20)30317-2 PMC7134694

[13] Ye Z, Wang Y, Colunga-Lozano LE Efficacy and safety of corticosteroids in COVID-19 based on evidence for COVID-19, other coronavirus infections, influenza, community-acquired pneumonia and acute respiratory distress syndrome: a systematic review and meta-analysis. CMAJ 192(27), E755–E767 (2020). 10.1503/cmaj.200645 PMC7828900 32409522

[14] Siddiqi HK, Mehra MR. COVID-19 illness in native and immunosuppressed states: a clinical-therapeutic staging proposal. J. Heart Lung Transplant. 39(5), 405–407 (2020). 32362390 10.1016/j.healun.2020.03.012 PMC7118652

[15] Azkur AK, Akdis M, Azkur D Immune response to SARS-CoV-2 and mechanisms of immunopathological changes in COVID-19. Allergy 75(7), 1564–1581 (2020). 32396996 10.1111/all.14364 PMC7272948

[16] Meduri GU, Annane D, Chrousos GP, Marik PE, Sinclair SE. Activation and regulation of systemic inflammation in ARDS: rationale for prolonged glucocorticoid therapy. Chest 136(6), 1631–1643 (2009). 19801579 10.1378/chest.08-2408

[17] Steinberg KP, Hudson LD, Goodman RB Efficacy and safety of corticosteroids for persistent acute respiratory distress syndrome. N. Engl. J. Med. 354(16), 1671–1684 (2006). 16625008 10.1056/NEJMoa051693

[18] RECOVERY Collaborative Group, Horby P, Lim WS Dexamethasone in hospitalized patients with COVID-19 – preliminary report. N. Engl. J. Med. (2020) (Epub ahead of print). 10.1056/NEJMoa2021436 PMC7383595 32678530

[19] Wang Y, Jiang W, He Q Early, low-dose and short-term application of corticosteroid treatment in patients with severe COVID-19 pneumonia: single-center experience from Wuhan, China. medRxiv (2020) (Epub ahead of print).

[20] Fadel R, Morrison AR, Vahia A Early short course corticosteroids in hospitalized patients with COVID-19. Clin. Infect. Dis. (2020) (Epub ahead of print). 10.1093/cid/ciaa601 PMC7314133 32427279

[21] Group TWHOREA for C-19 T (REACT) W. Association between administration of systemic corticosteroids and mortality among critically Ill patients with COVID-19: a meta-analysis. JAMA 324(13), 1330–1341 (2020). 32876694 10.1001/jama.2020.17023 PMC7489434

[22] COVID-19 Treatment Guidelines Panel. Coronavirus Disease 2019 (COVID-19) Treatment Guidelines. National Institutes of Health. http://www.covid19treatmentguidelines.nih.gov/34003615

[23] Adarsh Bhimraj A, Morgan RL, Hirsch Shumaker A Infectious Diseases Society of America Guidelines on the treatment and management of patients with COVID-19 (2020). http://www.idsociety.org/COVID19guidelines 10.1093/cid/ciaa478 PMC7197612 32338708

[24] Shang Y, Liu T, Wei Y Scoring systems for predicting mortality for severe patients with COVID-19. EClinicalMedicine 24, 100426 (2020). 32766541 10.1016/j.eclinm.2020.100426 PMC7332889

[25] Wu C, Chen X, Cai Y Risk factors associated with acute respiratory distress syndrome and death in patients with coronavirus disease 2019 pneumonia in Wuhan, China. JAMA Intern. Med. 180(7), 934–943 (2020). 32167524 10.1001/jamainternmed.2020.0994 PMC7070509

[26] Alqahtani JS, Oyelade T, Aldhahir AM Prevalence, severity and mortality associated with COPD and smoking in patients with COVID-19: a rapid systematic review and meta-analysis. PLoS ONE 15(5), e0233147 (2020). 32392262 10.1371/journal.pone.0233147 PMC7213702

[27] Halpin DMG, Singh D, Hadfield RM. Inhaled corticosteroids and COVID-19: a systematic review and clinical perspective. Eur. Respir. J. 55(5), 2001009 (2020). 32341100 10.1183/13993003.01009-2020 PMC7236828

[28] Yamaya M, Nishimura H, Deng X Inhibitory effects of glycopyrronium, formoterol, and budesonide on coronavirus HCoV-229E replication and cytokine production by primary cultures of human nasal and tracheal epithelial cells. Respir. Investig. 58(3), 155–168 (2020). 10.1016/j.resinv.2019.12.005 PMC7102607 32094077

[29] Iwabuchi K, Yoshie K, Kurakami Y, Takahashi K, Kato Y, Morishima T. Therapeutic potential of ciclesonide inahalation for COVID-19 pneumonia: report of three cases. J. Infect. Chemother. 26(6), 625–632 (2020). 32362440 10.1016/j.jiac.2020.04.007 PMC7161498

[30] Rockx B, Kuiken T, Herfst S Comparative pathogenesis of COVID-19, MERS, and SARS in a nonhuman primate model. Science 368(6494), 1012–1015 (2020). 32303590 10.1126/science.abb7314 PMC7164679

